# Out-of-Pocket, Catastrophic Health Expenditure and Distress Financing on Non-Communicable Diseases in India: A Systematic Review with Meta-Analysis

**DOI:** 10.31557/APJCP.2021.22.3.671

**Published:** 2021-03

**Authors:** Anushikha Dhankhar, Ranjeeta Kumari, Yogesh A Bahurupi

**Affiliations:** *Department of Community and Family Medicine, All India Institute of Medical Sciences, Rishikesh, India.*

**Keywords:** Out-of-pocket expenditure, catastrophic health expenditure, distress financing, NCDs, cancer, India

## Abstract

**Objective:**

The aim of this systematic review is to determine pooled estimates of out-of-pocket (OOPE) and catastrophic health expenditure (CHE), correlates of CHE, and most common modes of distress financing on the treatment of selected non-communicable disease (cancer) among adults in India.

**Methods:**

PubMed, Scopus and Embase were searched for eligible studies using strict inclusion and exclusion criteria. Data was extracted and pooled estimates using random effects model of meta-analysis were determined for different types of costs. Forest plots were created and heterogeneity among studies was checked.

**Results:**

The pooled estimate of direct OOPE on inpatient and outpatient cancer care were 83396.07 INR (4405.96 USD) (95% CI = 44591.05-122202.0) and 2653.12 (140.17 USD) INR (95% CI = -251.28-5557.53), respectively, total direct OOPE was 47138.95 INR (2490.43 USD) (95% CI = 37589.43-56690.74), indirect OOPE was 11908.50 INR (629.15 USD) (95% CI=-5909.33-29726.31) and proportion of individuals facing CHE was 62.7%. However, high heterogeneity was observed among the studies. Savings, income, borrowing money and sale of assets were the most common modes of distress financing for cancer treatment.

**Conclusion:**

Income- and treatment-related cancer policies are needed to address the evidently high and unaffordable cancer treatment cost. Economic studies are needed for estimating all types of costs using standardised definitions and tools for precise estimates. Robust cancer database/registries and programs focusing on affordable cancer care can reduce the economic burden and prevent impoverishment.

## Introduction

Non-communicable diseases (NCDs) are long duration (chronic) diseases, which result from a combination of factors, such as physiology, genetics, lifestyle-related behaviour and environment (WHO, 2018). Annually, 71% (41 million) of all global deaths are attributed to them (WHO, 2018). Among all, cancer has been reported to be the second leading cause of mortality globally, accounting for an estimated 9.6 million global deaths in the year 2018 (WHO, 2019). Cancer-related deaths range from 11.6% to 67.2% in India (Nethan et al., 2017). 

In India, the total number of most common cancers diagnosed in the year 2018 was 168,122 which includes oral, breast and cervical cancer (CBHI, 2019). Owing to its chronic nature and expensive treatment, out-of-pocket expenditure (OOPE) is the highest for cancer. In India, the insurance coverage is limited and health expenditures are not covered for approximately 80% of the population (Government of India, NSSO, 2020). Therefore, the treatment cost often results in financial catastrophe and distress compelling a household to resort to alternative ways of financing, which pushes the household in a deeper financial debt (WHO, 2010). Owing to lack of finances, knowledge and technological assistance, cancer patients and their caregivers, especially those who belong to the lower socioeconomic group, face significant financial and emotional struggle (Vashistha et al., 2019). 

A comprehensive evidence on out-of-pocket and catastrophic health expenditure (CHE) due to cancer is sparse (Kastor and Mohanty, 2018). Previously, the economic impact of cancer was estimated in systematic reviews assessing the overall economic burden of NCDs in Southeast Asia (Rijal et al., 2018) and in India (Mahal et al., 2010). Rijal et al., (2018) searched Medline and Embase to select quantitative studies published between 2000 and 2016 and conducted in Southeast Asia. Mahal et al., (2010) published a comprehensive discussion paper on economic implication of NCDs including cancer in India. In another review conducted by Vashistha et al., (2019), it was observed that the quality of life of caregivers of cancer patients reduces significantly. Quality of life was further lowered with increase in duration of care, especially for lower income households. This further brings the need to emphasise studying the economic burden of cancer in order to determine the extent to which mitigation of such a burden is required. Thecurrent systematic review aims to answer the following research questions:

1. What are the pooled estimates for OOPE on inpatient and outpatient care and the proportion of individuals facing CHE due to the treatment of selected non-communicable disease (cancer) among adults in India?

2. What are the various modes of distressed health financing (DHF) adopted by individuals for the treatment of cancer in India?

3. What are the factors associated with CHE faced due to the treatment of cancer among adults in India?

## Materials and Methods

The current systematic review is registered with PROSPERO (Registration number: CRD42020209497) and is reported following the MOOSE guidelines (Stroup et al, 2000). 

*Criteria for selection of studies*

All observational studies and government survey reports conducted among Indian adults (more than 18 years old, both males and females) suffering from cancer and published between January 2011 and July 2020 were included in the review. Newsletters, commentaries, editorials, studies conducted among cancer patients less than 18 years old or outside India and published before the year 2011 were excluded from the review.

*Search strategy*

Three bibliographic databases (PubMed, Embase, and Scopus) and government websites were searched for relevant studies and reports. Search terms and keywords based on population and exposure and those suggested by advanced database search operators were used to find relevant studies (supplementary Table 1). Boolean operators, such as “AND”, “OR” and “NOT” were used for refining the search strategy. 

*Screening Process*

After removing duplicate studies, titles and abstracts were screened independently by two reviewers using a piloted study screening tool. Subsequently, full-text screening of the selected studies was done and eligible studies were included in the review. Any discrepancies among reviewers were discussed with the third reviewer and resolved through consensus. 

*Data extraction*

Data extraction was performed by the first and second reviewer independently using a modifiable pre-designed data extraction form and a sample of data was cross-checked by the third reviewer. All OOPE was recorded in the Indian currency (INR) and US dollars (USD) and final expenditure was computed for the year 2020 as baseline. The ‘Campbell and Cochrane Economics Methods Group (CCEMG) - Evidence for Policy and Practice Information (EPPI) - Centre Cost Converter’ (v.1.6 last update: 29 April 2019) was used for cost conversions (Shemilt et al., 2019). If the year of measuring OOPE was not mentioned, the year before study publication was taken as the reference year for respective cost conversions. Outcomes, such as proportion of individuals facing CHE, modes of DHF and factors associated with CHE were also recorded. 

*Risk of bias assessment*

The study quality and risk of bias in the studies was checked using the Appraisal tool for Cross-sectional studies (AXIS) tool, which was modified based on the requirement of the current review (supplementary file) (Dowens et al., 2016). The minimum and maximum score that could be allotted to each study were 0 and 20, respectively. Studies with a score of 16 or above (80%), 12-15 (60-75%) or and less than 12 (<60%) were considered to be of high, moderate and satisfactory quality, respectively. 

*Data Analysis*

Narrative synthesis of data was undertaken for all the included studies. I^2^ test was applied to check heterogeneity among the studies while conducting meta-analysis using the Comprehensive Meta-Analysis software (Higgins et al, 2019). In case of high heterogeneity, random effect model was used as the tau-squared value was non-zero. The pooled estimates were reported along with the I^2^ values with 95% confidence interval (CI). 

*Outcome measures*

The following outcome measures were included in this review:

i. Direct OOPE, which comprised of the consultant charges, charges for diagnostic tests, amount spent on medicines and medical appliances, charges during stay at hospital, food, accommodation and transportation charges.

ii. Indirect OOPE, which included wage loss of the patient and the caretaker

iii. CHE, that is, when OOPE was more than 10% of total consumption expenditure or 40% of total non-consumption expenditure of the household. 

iv. Modes of DHF, that is, alternative ways a household adopts to pay for cancer treatment. 

## Results

After searching all accessible databases, a total of 9607 records were identified. After de-duplication, title and abstracts were screened for 8895 records. Out of these, 61 records were identified for full-text screening. After following strict inclusion and exclusion criteria, 21 studies were found eligible to be included in the systematic review. Two records were identified after manual searching of the references of the selected studies. A total of 23 studies were included and out of these, 15 records were included in the meta-analysis. 

**Figure 1:**
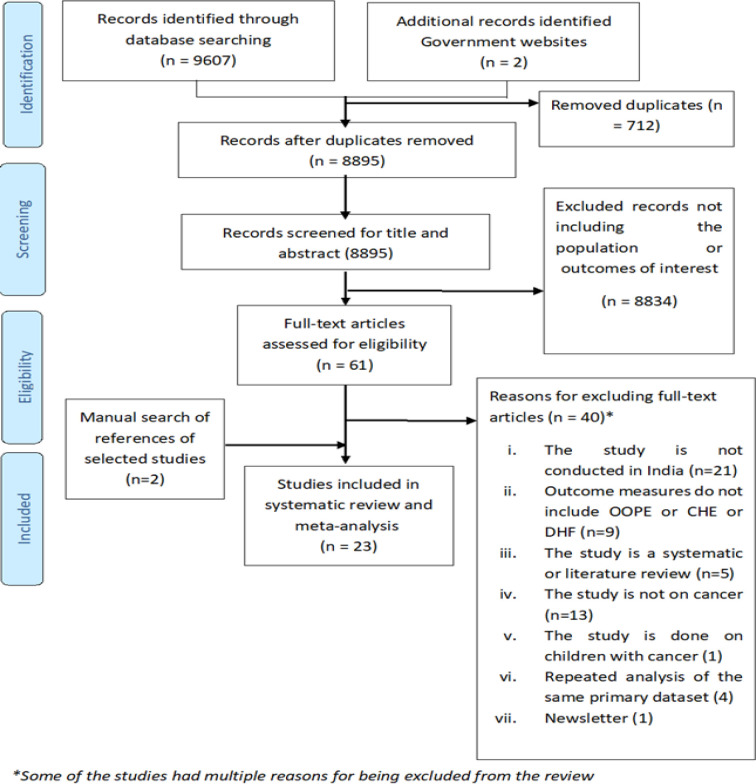
Prisma Flow Diagram for Selection of Eligible Studies

Figure 1 shows the steps followed for study selection based on the PRISMA guidelines (Moher et al., 2019).

*Overall study characteristics*

**Figure 2.**
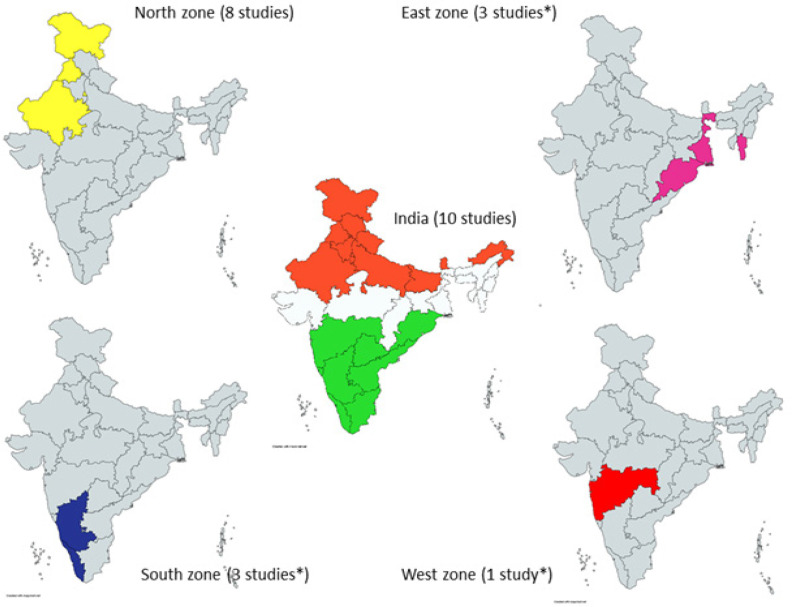
Geographical Distribution of Studies Included in the Review. *One of the studies was conducted in all the three zones. The maps shown above were created using a web-based tool, mapcharts.(16) They do not indicate the political administrative boundaries of India and are only for representation purposes. Zonal division is done as per the Zonal Councils of India

The current systematic review included 23 studies with data related to a total of 17,760 cancer patients. The overall study characteristics are described in Table 1. Figure 2 represents the geographical distribution of included studies.

*Characteristics of individual studies included in the systematic review*

Out of all studies, 34.8% (n=8) studies were cross-sectional, 34.8% (n=8) were prospective studies and 30.4% studies were secondary analyses of pan-India cross-sectional survey data. Approximately 52.2% (n=12) studies were community-based (or analysed data from community-based surveys) while the remaining 47.3% (n=11) studies were done in a hospital setting (supplementary Table 2). 

Quality and risk of bias assessment of the studies included in the systematic review

**Figure 3:**
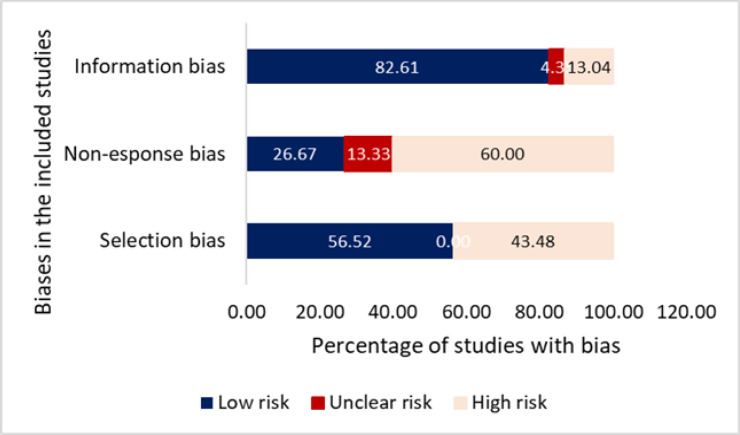
Percentage of Included Studies with Different Risks of Bias

Out of the selected 23 articles, 43.48% articles were found to be of high quality, 47.83% of moderate quality and 8.69% of satisfactory quality (supplementary table 3). Approximately 43.48% of the studies (n=10) had a high risk of selection bias, 60% (n=9) to had high risk of non-response bias and 13.04% (n=3) had a high risk of information bias (supplementary Table 4, Figure 3). Among all, 82.6% studies used validated tools to measure outcomes and risk factors. 

OOPE incurred on cancer treatment in India

A total of 12 (52.2%) studies measured direct and five out of these (41.6% of 12 studies) measured indirect OOPE on cancer treatment (supplementary Table 5). 

**Figure 4:**
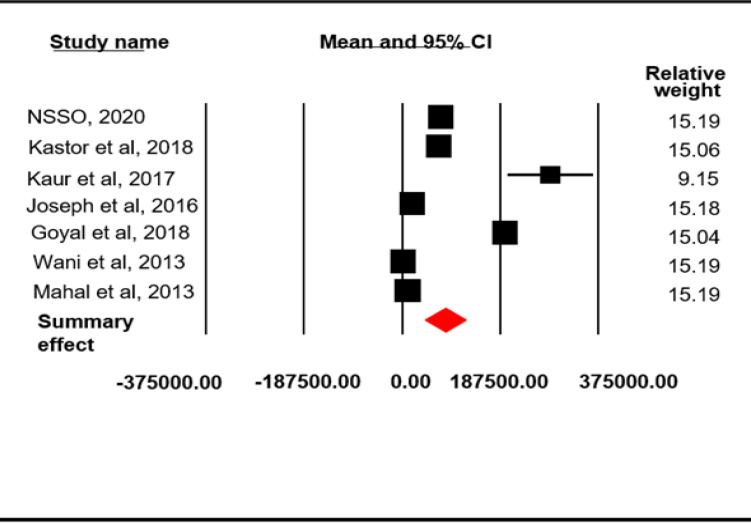
Meta-Analysis Results for Direct OOPE on Inpatient Cancer Care: Random Effect Model

**Figure 5:**
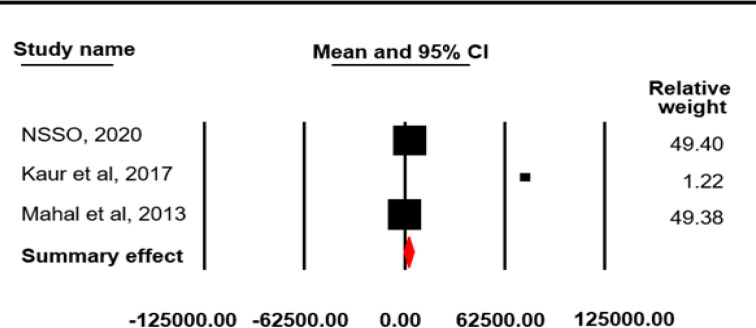
Meta-Analysis Results for Direct OOPE on Outpatient Care: Random Effect Model

**Figure 6:**
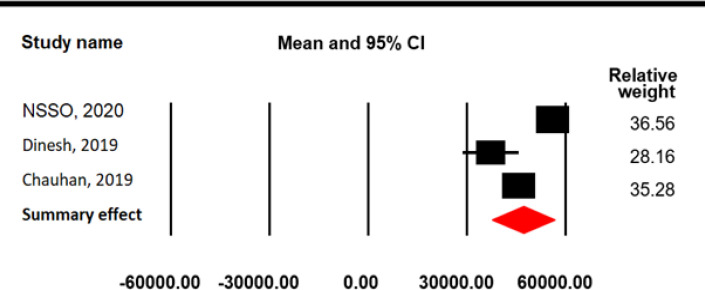
Meta-Analysis Results for Total Direct OOPE on Inpatient and Outpatient Care: Random Effect Model

The reference period for estimating direct OOPE on inpatient cancer care was 365 days. The pooled mean for direct OOPE on inpatient cancer care was found to be 83396.07 INR (4405.96 USD) (95% CI=44591.05-122202.0) (Figure 4) with a high-level between-study heterogeneity (I^2^=99.98, p=0.0001). Among the selected studies, five reported direct OOPE on outpatient cancer care taking 15 days as reference period. The pooled mean direct OOPE on outpatient cancer care was found to be 2653.12 INR (140.17 USD) (95% CI=-251.28-5557.53; SE=1481.56 INR; I^2^=99.94, p=0.0001) (Figure 5). The pooled mean total direct OOPE on both inpatient and outpatient cancer care was 47138.95 INR (2490.43 USD) (95% CI=37589.43-56690.74; SE=4872.88 INR; I^2^ = 96.88, p = 0.0001) (Figure 6).

**Figure 7:**
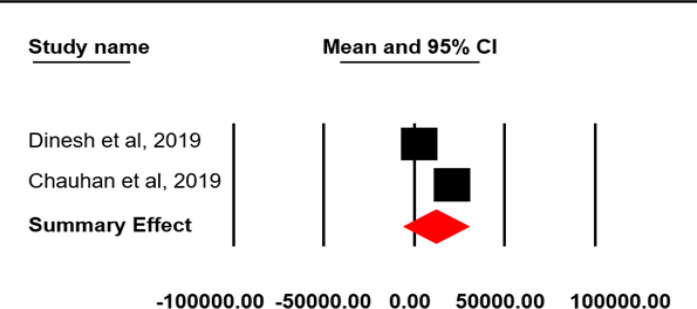
Meta-Analysis Results for Indirect OOPE in Cancer Care: Random Effect Model

Only five of the included studies reported indirect cost incurred on cancer care and its pooled estimate was found to be 11908.50 INR (629.15 USD) (95% CI=-5909.33-29726.31; SE=9090.89 INR; I2=99.84; p=0.0001) (Figure 7). 

*Catastrophic health expenditure (CHE) on cancer treatment in India*

**Figure 8:**
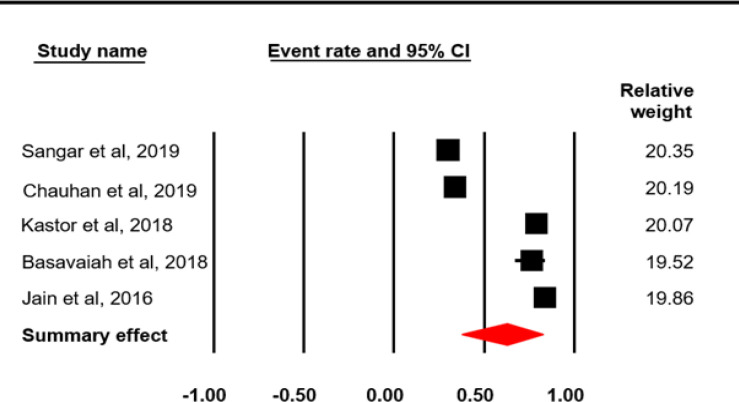
Meta-Analysis Results for Proportion of Cancer Patients Facing CHE: Random Effect Model

The reported proportion of cancer patients suffering financial catastrophe varied across the studies (supplementary Table 6). The pooled event rate of individuals facing catastrophic health expenditure availing cancer care was found to be 0.627 (62.7%; I^2^ = 98.60; p = 0.0001) (Figure 8).

*Modes of distress financing for cancer treatment in India*

Table 2 describes the modes of DHF adopted by cancer patients while undergoing treatment. The findings reveal that savings or income, borrowing money from friends or social connections, and selling property or assets are most commonly adopted modes. One of the studies also revealed discontinuing treatment and continuing to live with cancer as one of the coping strategies. 

*Correlates of Catastrophic health expenditure on cancer care in India*

Out of the six studies addressing CHE, five had analysed its predictors which were not comparable. Therefore, meta-analysis to determine the effect of various factors associated with CHE could not be conducted. 

Sangar et al., (2019) found that headcount for CHE was more among richer sections as compared to the poorer ones. Chauhan et al., (2018) found that the odds of CHE were higher for lower income quartile patients (OR: 5.6, 95% CI: 2.6–12.4, p-value: <0.001) than those in the highest income quartile. Also, the patients undergoing intensity-modulated radiotherapy (IMRT) faced higher odds of incurring CHE (OR=3.516; 95%CI=1.61-7.66, p-value<0.002) as compared to those receiving two-dimensional radiotherapy (2-DRT). Rajpal et al., (2018) found in their study that 36.3% (public healthcare facilities) and 33.7% (private healthcare facilities) of cancer patients were spending more than 10% of their annual per capita household expenditure (PCHE). Over 50% of the cancer patients from low-income households were reported to be spending more than 10% and 20% percent of PCHE. In contrast, around 26% of richer households were reported spending more than 10% and 20% of their annual income on cancer treatment. 

In another study by Jain and Mukherjee (2016), lower income households were reported to be 39.38 times and middle income households to be 5.79 times more likely to face CHE as compared to higher income households. Cancer patients utilising private facilities were reported to be 62.2 times more likely to face CHE than those seeking treatment at public healthcare facilities. Also, patients with second or higher stage of cancer were reported to be at 16.29 times higher risk of facing CHE (Jain and Mukherjee, 2016). Tripathy et al., (2016) found that the odds of facing CHE were 12.2 times for cancer treatment as compared to that for a communicable disease. 

**Table 1:** Overall Characteristics of the Selected Studies

Characteristics of the studies	Number of studies (%)
Year of Publication	
2011-2015	14 (63.64%)
2016-2020	8 (36.36%)
Year of cost estimation	
1995-2005	2 (9.09%)
2006-2015	16 (72.73%)
2016-2020	4 (18.18%)
Location (11)	
North zone	7 (31.82%)
East zone	2 (9.09%)
West zone	1 (4.54%)
South zone	3 (13.64%)
Pan-India	9 (40.91%)
Outcomes	
Studies on OOPE	17 (77.28%)
Studies on financial catastrophe	6 (27.27%)
Studies on distress financing	5 (22.73%)
Type of cancer	
General (not specified)	13 (59.1%)
Specific (e.g., breast, oral, cervical cancer, etc.)	9 (40.9%)
Sample size range (8 - 11,112)	
0-500	19
501-1,000	3
>1,000	1

**Table 2:** Result of Meta-Analysis for Estimating OOPE and CHE

Direct OOPE on inpatient cancer care in India					
Study Name	Mean	Standard Error	Variance	Lower limit	Upper limit
NSSO, 2020	75689.09	2.19	4.82	75684.8	75693.39
Kastor et al, 2018	70763.17	4778.85	222837415.4	61396.79	80129.55
Kaur et al, 2017	284688.06	41253.02	1701811739	203833.63	365542.5
Joseph et al, 2016	19925.98	946.59	896038.77	18070.69	21781.27
Goyal et al, 2014	198198.58	5063.85	25642546.44	188273.62	208123.54
Wani et al, 2013	3430.85	75.48	5697.83	3282.9	3578.8
Mahal et al, 2013	12061.32	365.78	133792.6	11344.41	12778.23
Summary effect	83396.52	19799.08	392003454.3	44591.05	122202
Direct OOPE on outpatient cancer care in India					
Study Name	Mean	Standard Error	Variance	Lower limit	Upper limit
NSSO, 2020	3238.92	4.75	22.56	3229.61	3248.23
Kaur et al, 2017	75282.16	13235.45	175177246.8	49341.15	101223.17
Mahal et al, 2013	268.73	49.38	2438.27	171.95	365.51
Summary effect	2653.13	1481.86	2195924.37	-251.28	555.53
Total direct OOPE on cancer care in India					
Study Name	Mean	Standard Error	Variance	Lower limit	Upper limit
NSSO, 2020	56105.95	1.99	3.98	56102.04	56109.86
Dinesh et al, 2019	37242.05	4401.8	19375851.15	28614.68	45869.42
Chauhan et al, 2019	45749.05	1531.11	2344294.11	42748.63	48750.47
Summary effect	47140.08	4872.88	23744905.93	37589.43	56690.74
Indirect OOPE on cancer care in India					
Study Name	Mean	Standard Error	Variance	Lower limit	Upper limit
Dinesh et al, 2019	2802.88	733.34	537790.49	1365.56	4240.2
Chauhan et al, 2019	20984.69	53.32	2843.39	20880.18	21089.2
Summary effect	11908.5	9090.89	82644337.3	-5909.33	29726.31
Proportion of cancer patients facing CHE in India					
Study name	Event rate		Lower Limit		Upper Limit
Sangar et al, 2019	0.3		0.292		0.309
Chauhan et al, 2019	0.34		0.296		0.387
Kastor et al, 2018	0.79				0.831
Basavaiah et al, 2018			0.671		0.839
Jain et al, 2016	0.84		0.786		0.883
Summary effect	0.627		0.378		0.823

**Table 3:** Modes of Distress Financing and Coping Strategies for High OOP Payments on Cancer Care in India

Reference article	Reported modes of distress financing	Proportion of cancer patients
Alexander et al, 2019	Savings Mortgaging or selling assets High-interest loan or discontinuation of therapy Discontinued treatment	28% 30% 13% 3%
Jain and Mukherjee, 2016	Borrowed money at low interest (0-15% p.a.) rates Used social nets that is monetary assistance form relatives and friends Savings Used financial assets that is shares, mutual funds and gold Delayed payments of pre-existing loans Sold economic productive assets Renting out Delayed payment of bills Pawned jewellery Borrowed money at a high interest (≥15%) rate took credit from local shop (15.8%), financing by aid (govt/private) (14%)	84.60% 74.70% 74.20% 53.40% 48.90% 41.20% 33% 19.90% 16.70% 15.80% 15.80% 14%
Joe, 2015	Income/savings Borrowing Contributions from relatives or friends sale of assets	-

## Discussion

The current systematic review included 23 eligible studies with an overall picture of OOPE incurred, CHE faced and modes of distress financing adopted by cancer patients in India. The studies varied in design, sampling strategy, and reporting of outcome measures rendering themselves at risk of including different types of biases. Selection bias was the most commonly encountered bias among studies followed by non-response bias. Information on response rate, reporting standard deviation or confidence intervals and median costs was lacking in some of the studies given the fact that economic studies often show skewed distribution. In the current systematic review where studies showed highly variable sample sizes, a normal distribution of outcome measures was less likely. Moreover, the studies also vary in quality due to inconsistent definitions and lack of reference group.

The pooled mean direct cost for inpatient cancer treatment is significantly high (83345.026 INR; p=0.0001) as compared to other costs. A similar finding was also reported in a systematic review conducted by Rijal et al., (2018). Another systematic review on NCDs (including cancer) also reported that the highest proportion of OOPE is attributable to direct medical cost, especially medications, for the treatment of cancer. The pooled mean OOPE on outpatient cancer care in a reference period of 15 days was found to be 2653.12 (1481.87) INR. 

Through these findings, it is evident that the OOPE on cancer care is high and unaffordable, which is also reported by all of the included studies. However, individual study cost estimates were highly variable and heterogeneity was evident from the meta-analysis results. One of the possible reasons could be attributed to the differences in study designs. Cross-sectional studies record the costs at one point of time while prospective studies give an elaborate picture. Those with large sample size are likely to report more precise outcome measures as compared to small-scale studies with non-representative sample population. Another reason could be the variation in operational definitions of direct OOPE on cancer care and type of healthcare facility utilised resulting in significantly different results. 

Very few studies measured indirect cost associated with cancer treatment. The pooled mean indirect OOPE was also very high [11908.53 (9090.89) INR], however, the result was not statistically significant (p=0.190). It is evident that indirect expenditure in the form of wage loss of the patient and the caregiver is high. The employment sector being largely informal, health seeking behaviour may also be guided by loss of wages on daily basis due to cancer treatment. Inconsistencies in defining indirect OOPE among the included studies was also seen across these studies. 

The pooled estimate of the percentage of cancer patients facing CHE was 62.7%, which is drastically high. The reason for such a high proportion of cancer patients experiencing CHE may be attributed to different cut-off levels to determine CHE across studies, differences in incomes and socioeconomic status of cancer patients, cost of public and private healthcare facilities, cost of various treatment modalities and advancing stage of cancer (Rajpal et al., 2-18; Jain and Mukherjee, 2018). The possibility of poor sections of the society to be financially drained and henceforth, not seeking medical care is also high. This was evident from the study conducted in Karnataka (Alexander et al., 2019) which reported 3% of the cancer patients who had discontinued treatment due to high OOPE and associated financial catastrophe. 

The most common modes of DHF for cancer care are savings, borrowing money and sale of assets. Similar findings have been reported by a previously done systematic review (Rijal et al., 2018). The most dreadful coping strategy was discontinuing treatment. Health insurance being an unpopular practice in India, the dependency on alternative means of financing makes the households unable to cope with similar health-related incidents in future. After more than one year of the launch of Ayushman Bharat under the Pradhan Mantri Jan Aarogya Yojana, no study has been conducted so far to evaluate the current status of healthcare financing in cancer, especially among the vulnerable families who are beneficiaries of the program. 

Only a few out of the selected 23 studies have identified the possible predictors of CHE among cancer patients. Such a gap in existing data may be due to poorly functioning healthcare management systems and absence of population-based cancer registries in India. This results in underreporting of cancer cases and underestimation of overall economic impact of cancer. 

To monitor the level of financial protection, WHO recommends using incidence of CHE among patients seeking health care services. Most of the studies included in this review addressed the proportion of individuals facing CHE at one point of time. In addition, half of the studies were secondary analyses of cross-sectional surveys, failing to provide evidence on temporal relationship between CHE and its predictors. 

As per the findings of current systematic review, only a handful of primary studies have been conducted on the economics of cancer in the country, especially when available literature provides strong evidence that cancer has the highest economic burden among all NCDs, both on cancer patients and health systems. The current knowledge gap of its impact subsequently slows down the already lagging preventive and curative health program implementation in the target populations to help financially challenged households. Hence, crucial information still lies in the hands of future robust research studies. Emphasising on the duration for which a household is under financial catastrophe is also important, especially in case of cancer where the treatment cost is high and the disease is often associated with recurrences and complications. 

The main limitation of our review is using only three databases for screening and identifying studies published after 2010. However, a thorough search of the references was also done to include any eligible studies in the review. Also, in most of the studies, the status of cancer was self-reported and only a few studies used diagnosed cancer as the inclusion criteria, rendering the current analysis to have possibly overestimated the costs. Besides this, recall bias in providing cost-related information in all the studies is another challenge that may have resulted in under-estimation of actual OOPE on cancer treatment after data pooling. Due to lack of availability of data on correlates of financial catastrophe in the included studies, the current review could not identify the risk factors or predictors associated with it. 

One of the strengths of the current systematic review is the use of a comprehensive, peer-reviewed and validated tool to assess the quality as well as risk of bias of the included studies. The review also provides pooled estimates in both Indian and US currencies allowing to extrapolate the pooled cost estimates to the current year (2020) and increase comparability with international studies. It was able to highlight the seriousness of expenditures incurred by cancer patients (both direct and indirect cost) while seeking cancer treatment. The costs are unaffordable for people already existing below the poverty line. This brings the need of special income- or treatment-related policy and evidence-informed nationally tailored prepayment mechanisms with consideration of patients suffering from cancer, especially for those from the informal sector and low socioeconomic status. Through the current systematic review, the gaps in data availability, poor data reporting and high variability among studies assessing the cost could also be highlighted. These findings reveal the need of robust research required in adequately measuring cost-related data with reference/comparator group along with optimal study design and universal definitions to measure outcomes to prevent variations in cost estimates. It also emphasises on the need of preventive cancer strategies and early detection of cancer. A cancer registry to record all cost-related data could be incorporated in the present healthcare system to identify individuals likely to face financial distress to whom subsidised care could be provided. An interventionist mass insurance policy as a joint venture between the government and private insurance companies for cost sharing can potentially make cancer care affordable. 

## Author Contribution Statement

The authors confirm contribution to the paper as follows:

Study conception and design: Anushikha Dhankhar, Ranjeeta Kumari; data collection: Anushikha Dhankhar, Yogesh A Bahurupi; analysis and interpretation of results: Anushikha Dhankhar, Ranjeeta Kumari, Yogesh A Bahurupi; draft manuscript preparation: Anushikha Dhankhar. All authors reviewed the results and approved the final version of the manuscript. 
